# The Bitter Taste Receptor TAS2R16 Achieves High Specificity and Accommodates Diverse Glycoside Ligands by using a Two-faced Binding Pocket

**DOI:** 10.1038/s41598-017-07256-y

**Published:** 2017-08-10

**Authors:** Anu Thomas, Chidananda Sulli, Edgar Davidson, Eli Berdougo, Morganne Phillips, Bridget A. Puffer, Cheryl Paes, Benjamin J. Doranz, Joseph B. Rucker

**Affiliations:** Integral Molecular, Inc., 3711 Market St, Suite 900, Philadelphia, PA 19104 USA

## Abstract

Although bitter taste receptors (TAS2Rs) are important for human health, little is known of the determinants of ligand specificity. TAS2Rs such as TAS2R16 help define gustatory perception and dietary preferences that ultimately influence human health and disease. Each TAS2R must accommodate a broad diversity of chemical structures while simultaneously achieving high specificity so that diverse bitter toxins can be detected without all foods tasting bitter. However, how these G protein-coupled receptors achieve this balance is poorly understood. Here we used a comprehensive mutation library of human TAS2R16 to map its interactions with existing and novel agonists. We identified 13 TAS2R16 residues that contribute to ligand specificity and 38 residues whose mutation eliminated signal transduction by all ligands, providing a comprehensive assessment of how this GPCR binds and signals. Our data suggest a model in which hydrophobic residues on TM3 and TM7 form a broad ligand-binding pocket that can accommodate the diverse structural features of β-glycoside ligands while still achieving high specificity.

## Introduction

Human bitter taste perception is mediated by the 25 members of the highly divergent TAS2R receptor family^1^. The TAS2Rs are expressed in taste cells as well as cells in the respiratory and gastrointestinal tracts, and have evolved to detect the extraordinary diversity of bitter compounds that is naturally found in foods and toxins, translating that detection into gustatory perception via G protein-coupled signaling^2, 3^. The prototypical bitter taste receptor TAS2R16 is known to respond to ~30 different β-glucoside compounds^4–6, 30^ whose molecular scaffold consists of a D-glucose monosaccharide linked by an oxygen atom to a phenyl group. Many plants, including cruciferous vegetables such as broccoli and brussels sprouts, contain bitter β-glucosides such as salicin, sinigrin, arbutin, and amygdalin. Thus TAS2R16 specifically is believed to play a central role in determining human preference to eat or avoid such vegetables, important dietary choices that ultimately influence human health.

The ability to precisely perceive bitter taste through TAS2R receptor activation enables the selection of beneficial foods safe for ingestion and the avoidance of potential toxins^7^. However, the detection of bitter taste must accommodate the broad diversity of chemical structures found in nature while simultaneously being tuned to an optimal sensitivity and binding affinity such that not all food substances taste overwhelmingly bitter. Due to these requirements, TAS2R receptors have been subject to large evolutionary pressures, resulting in a substantial number of polymorphisms that shape daily choices that impact health, disease, and longevity^8^. For example, individual TAS2R polymorphisms appear to influence body mass index^9^, alcohol intake^10^, smoking^11^, compliance with medications^12^, and human lifespan^13^. Human TAS2R16 alone has at least 17 polymorphisms, including an allelic variant at amino acid 172 that is associated with a 2-fold decrease in sensitivity to β-glucosides and is linked to alcohol dependence^10, 14, 15^. Understanding how such polymorphisms influence the structure and function of TAS2R receptors could provide a mechanistic explanation for how TAS2R genotypes translate into human phenotypes and behaviors.

Human bitter taste receptors have evolved the ability to detect and respond to an enormous range of chemical classes such as β-glycosides, thioureas, and sesquiterpene lactones, with specific receptors able to respond to an array of related structures^5^. It has thus been challenging to explain how a TAS2R, or any GPCR, is able to recognize a specific chemical class while accommodating the chemical diversity within that class. Although the cognate ligands have been defined for a number of TAS2Rs, their mechanisms of interactions with receptor structures have not been well explored, in part because most TAS2R ligands bind extremely weakly with relatively low EC50 values (at µM to mM levels)^5, 16^. To date, our understanding of TAS2R ligand recognition has come primarily from mutational analyses of small subsets of residues or from *in silico* models based on crystal structures of distantly related proteins such as rhodopsin^6, 16–18^. Such studies broadly suggest a TAS2R ligand-binding pocket formed by several transmembrane (TM) domains (particularly TM3, TM5, TM6, and TM7^19^). No crystal structures of any TAS2R receptor currently exist, and co-crystal structures with such low affinity ligands present an even larger challenge. Thus, it remains unclear what structural mechanisms are used by these GPCRs to detect the enormous diversity of natural bitter compounds while simultaneously achieving high selectivity for specific types of molecules.

Our approach to answering this question has focused on a ligand structure–activity relationship analysis with a comprehensive library of single amino acid mutations covering all 291 residues of TAS2R16. We have used this approach previously to characterize the signal transduction mechanism of the GPCR CXCR4^20^. Our structure-function analysis of human TAS2R16 identified 13 residues that contribute to ligand-specific interaction and 38 whose mutation eliminated signal transduction by all ligands, providing a comprehensive assessment of how this GPCR binds and signals. We also provide molecular evidence for the plasticity of the TAS2R16 binding site and an explanation for the relatively broad specificity of the receptor.

The interaction requirements of the ligand-binding pocket residues of TAS2R16 enable us to propose a model for how this bitter taste receptor maintains broad reactivity yet high specificity so that it can detect diverse β-glycosides without all foods tasting bitter. Many of the critical residues identified are conserved among TAS2R family members, suggesting that the mechanisms used by TAS2R16 may also be more broadly applicable to other TAS2Rs.

## Results

### Identification of TAS2R16 residues critical for salicin-mediated signaling and cell surface trafficking

**Figure 1:**
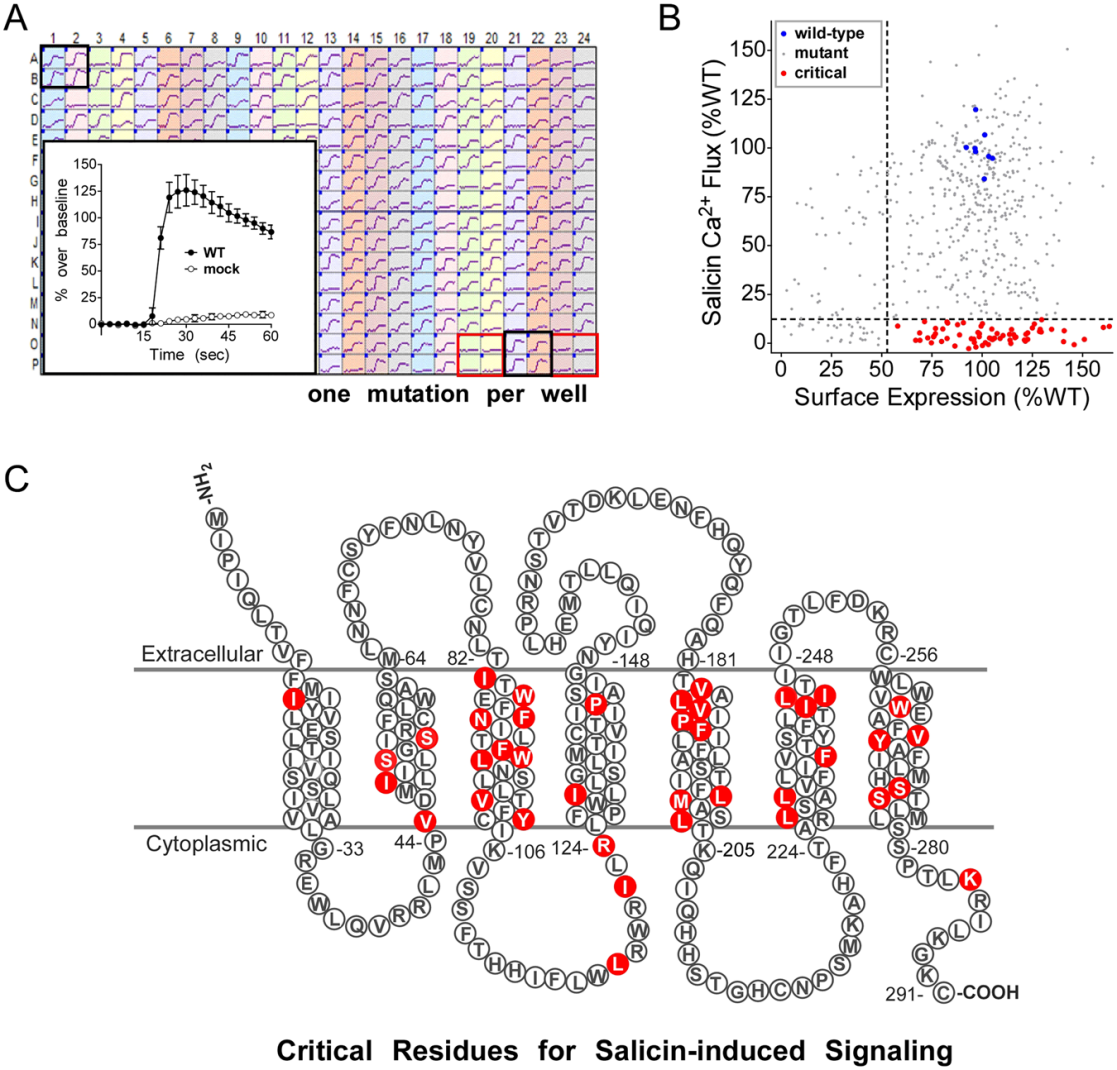
Identification of residues critical for salicin-dependent signaling. (**a**) Calcium traces obtained from a representative 384-well plate of HEK-293T cells transfected with the TAS2R16 mutation library and stimulated with 3 mM salicin. Each library plate contained 8 positive control wells used for normalization of Ca^2+^ flux measurements (wild-type TAS2R16; black boxes) and 8 negative controls wells (non-specific vector; red boxes). Individual traces for positive and negative control wells are shown in the inset (average+/− standard deviation). (**b**) Plot showing TAS2R16 salicin-induced Ca^2+^ flux as a function of cell surface expression (detected by immunofluorescent detection of an N-terminal FLAG tag) for each clone in the TAS2R16 mutation library. Values are normalized to wild-type, shown in blue. Critical clones marked in red displayed less than 12% (mean of negative controls +3*SD) of wild-type TAS2R16 Ca^2+^ flux activity and greater than 53% (mean of positive controls - 3*SD) of wild-type TAS2R16 cell surface expression. (**c**) Diagram of TAS2R16 showing the location of residues whose mutations eliminated Ca^2+^ flux for salicin (i.e. flux <12%; highlighted in red) and gave expression on the cell surface at >53% of wild-type. TMs were predicted using TMHMM Server v. 2.0.

To investigate the structural basis of bitter taste receptor activation and ligand selectivity, a comprehensive ‘shotgun mutagenesis’ mutation library of receptor variants^20, 21^ was created with a total of 573 individual mutant TAS2R16 clones, representing an average of 2 substitutions at each amino acid position (typically one conserved and one non-conserved substitution per position). The entire TAS2R16 mutation library was transfected into human HEK-293T cells in a 384-well array format (one clone per well) and evaluated for salicin-dependent activation measured by a calcium flux assay (Fig. 1a). Salicin is highly prevalent in plants and the best characterized natural ligand of TAS2R16, and Ca^2+^-flux signaling assays are commonly used to measure the function of TAS2R16 and other GPCRs, so this measurement represents the key function of the receptor. The addition of salicin to cells expressing wild-type TAS2R16, but not mock-transfected cells, resulted in robust receptor activation, measured as an increase in cellular fluorescence.

Each TAS2R16 variant was also independently assessed for full-length translation using a C-terminal V5 epitope tag and for surface expression using an N-terminal FLAG epitope tag (Figure S2a). Most mutant clones in the TAS2R16 library were fully translated (91% of clones were fully expressed at >50% of wild-type levels), were well-expressed on the cell surface (88% of clones successfully trafficked to the cell surface at >50% of wild-type levels), and encoded functional receptors (60% of clones signaled at >50% of wild-type levels).

At a total of 39 positions in TAS2R16, substitution resulted in significantly reduced activation by salicin without disrupting the surface trafficking of the receptor (Fig. 1b and Supplementary Table S1). Mapping of these critical residues onto a schematic of TAS2R16 revealed that 90% of residues (35 of 39) cluster within the TM domains, with the highest incidence in TM3 (9 residues) and TM5 (8 residues) (Fig. 1c). The predominance of critical residues in TM3 and TM5 is comparable to class A GPCRs, where these same helices undergo significant conformational changes upon activation^22–24^. TM1 and TM4 contained the fewest number of critical residues (1 and 2 residues respectively), consistent with the limited role of these helices in activation of class A GPCRs^23, 25^. For the extracellular loops (ECLs) we did not identify a single mutation that eliminated TAS2R16 activation by salicin, suggesting that the ECLs are not important for the interaction with salicin. While it is known that conformational changes in the TM domains of GPCRs are key contributors to signal transduction^24^, a comprehensive set of residues essential for the signaling of a TAS2R has never before been identified.

Only 17 TAS2R16 variants resulted in decreased surface trafficking while expressing at near wild-type levels, with most of these mutations (10 of 17) located in TM1 and TM2 (Figure S2b and Supplementary Table S2). The majority of these mutations (11 of 17) were substitutions to arginine at positions in the TMs and, predictably, they reduced or eliminated both surface trafficking and salicin-induced Ca^2+^ flux. Substitution N172K, in ECL2, was the only trafficking mutant that retained substantial signaling activity (83% of wild-type activity, with 48% of wild-type surface trafficking). Position 172 was also the only extracellular mutation that decreased surface expression. Interestingly, position 172 is a well-defined TAS2R16 polymorphic site, where the Asn-containing allele (N172) is associated with a 2-fold increased sensitivity to β-glucosides, including salicin^14, 15, 26–28^. Our data demonstrates that the N172 variant of this position expresses at the cell surface approximately 2-fold higher than the K172 variant, thus providing one potential molecular explanation for the K172 variant’s decreased sensitivity to β-glucosides and relation of this polymorphism to alcohol dependence^10, 14, 15^.

### Identification of novel TAS2R16 agonists

**Figure 2:**
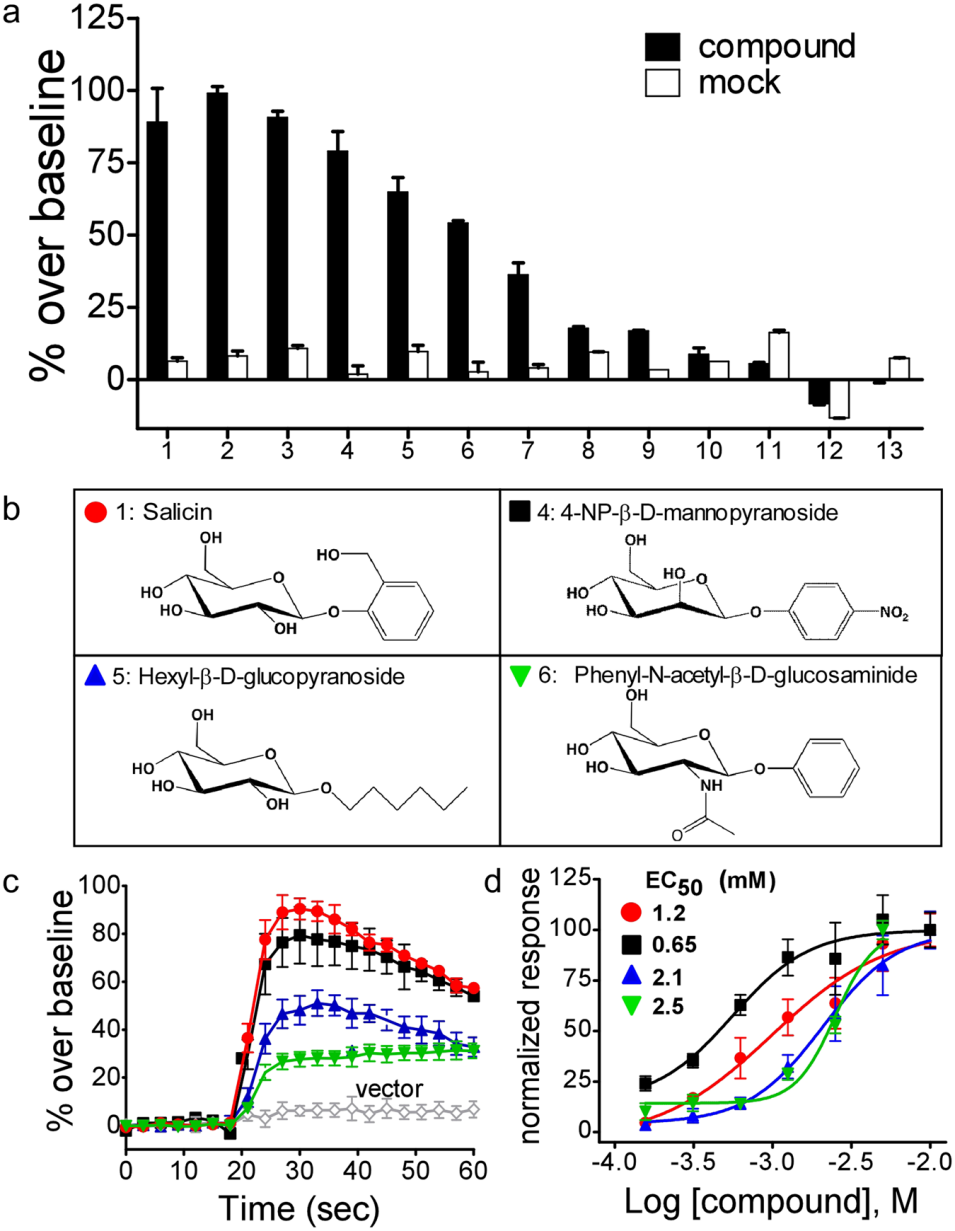
Identification of novel TAS2R16 agonists. (**a**) Salicin and 12 related compounds were screened at 10 mM (the highest level practical) for their ability to signal through TAS2R16. The compounds assayed were: 1, salicin; 2, phenyl-β-D-thioglucopyranoside; 3, phenyl-β-D-glucoside; 4, 4-nitrophenyl-β-D-mannopyranoside; 5, hexyl-β-D-glucoside; 6, phenyl-N-acetyl-β-D-glucosaminide; 7, sinigrin; 8, 2-naphthyl-β-D-glucopyranoside; 9, esculin; 10, methyl-β-D-glucopyranoside; 11, phenyl-α-D-glucopyranoside; 12, 1-O-phenyl-β-D-xyloside; 13, phenyl-β-D-galactopyranoside. (**b**) The structures of salicin and the three TAS2R16 agonists selected for further study. (**c**) Representative Ca^2+^ flux traces after addition of salicin or selected analogs. (**d**) Dose-response curves for salicin and selected analogs. The values for each ligand were normalized to the maximum % over baseline signal (defined as 100%), to highlight the differences in EC_50_s among the ligands. The EC_50_ of salicin was 1.2 mM, consistent with its reported value of 3 mM^4^. Error bars represent the standard deviation, n = 4–8 replicate points.

Bitter taste receptors need to respond to thousands of different chemical structures to detect the range of natural bitter and potentially toxic compounds present in the environment. To help define the TAS2R16 ligand structural features that confer specificity, we first tested the agonist activity of a panel of twelve readily available salicin analogs representing specific structural changes in the β-glucoside core constituents (R groups, glycosidic linkage, and monosaccharide units). Six of the twelve analogs demonstrated moderate to robust activation of TAS2R16 (Fig. 2a and Supplementary Fig. S1), and two of them have not been previously described as TAS2R16 agonists (phenyl-β-D-thioglucopyranoside and phenyl-N-acetyl-β-D-glucosaminide).

Three compounds were selected for additional characterization based on the diversity of their structures (Fig. 2b): 4-nitrophenyl-β-D-mannopyranoside (hereafter 4-NP-β-mannoside), hexyl-β-D-glucopyranoside (hexyl-glucoside), and phenyl-N-acetyl-β-D-glucosaminide (β-glucosaminide). Despite substantial differences in the monosaccharide and R groups among these three analogs and salicin, their activation of TAS2R16 varied by only a roughly four-fold difference in EC50 values (ranging from 0.65 mM to 2.5 mM) (Fig. 2c). Bitter taste is thought to have evolved, in part, to detect the sum total of potential toxic compounds (bitter tastants) in food, which would be aided by compounds having similar EC50 values for a given receptor^7, 29^.

The additional compounds were selected to provide different types and size of R group, a large substitution in the sugar moiety, and include a biologically important compound (4-nitrophenyl-β-D-mannopyranoside) that differs from salicin solely by the orientation of the 2′-OH group on the sugar. Although TAS2R16 has a number of restrictions on ligand recognition such as not responding to β-galactosides^4^, receptor activation by 4-NP-β-mannoside^30^ demonstrates that substrate detection is not restricted to β-glucosides, suggesting that TAS2R16 has a broad role in bitter detection. Its ability to detect phenolic β-mannosides and more complex phenolic β-glucosides of plants^31, 32^, suggests that TAS2R16 may be an important determinant of herbivore food selection.

### Identification of TAS2R16 residues that determine ligand selectivity

**Figure 3:**
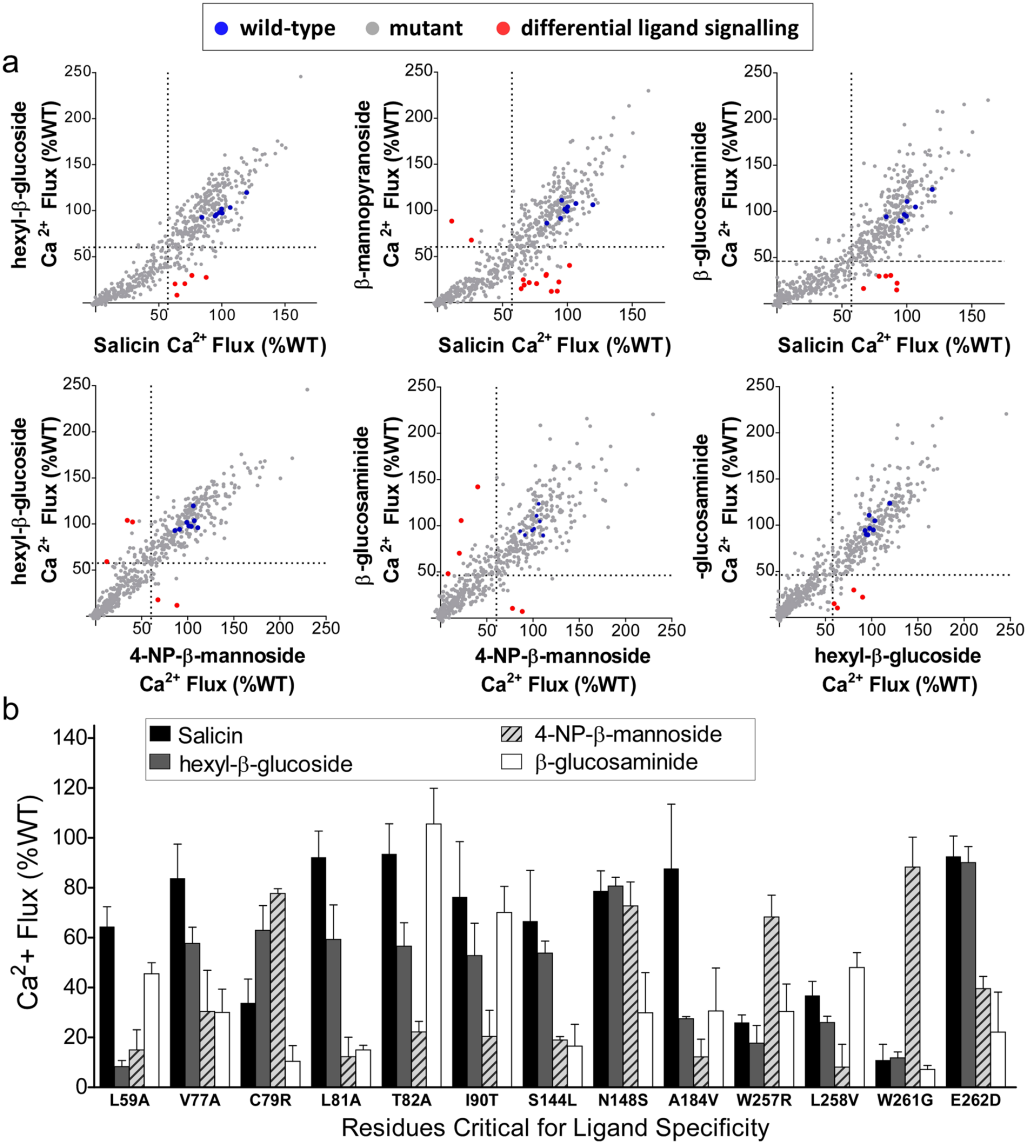
Identification of ligand-specific structures. (**a)** Each of the four TAS2R16 ligands selected was tested against the entire TAS2R16 mutation library (573 individual mutations, n = 2–3) and values derived for each are compared. Concentrations chosen for mutation library screening were approximately 2 to 3 times higher than the EC_50_ value for each ligand to maximize the sensitivity of detecting decreases in signaling due to the mutation in each clone (3 mM salicin, 5 mM hexyl-β-glucoside, 1.6 mM 4-NP-β-mannoside, and 10 mM β-glucosaminide). Cutoffs of (100-3*SD), derived from wild-type positive control wells for each ligand, were used to identify clones that showed high Ca^2+^ flux for one ligand but low Ca^2+^ flux for the other (57% for salicin, 58% for hexyl-glucoside, 53% for 4-NP-β-mannoside, and 46% for β-glucosaminide). Of these critical residues, mutants that signaled 2.5-fold higher or lower were identified as the most significant (shown in red). Average values obtained from wild-type controls for each ligand are shown in blue. **(b)** Ca^2+^ flux activities are shown for clones expressing mutations that resulted in a ≥ 2.5-fold difference between Ca^2+^ flux activities for any two ligands.

**Figure 4:**
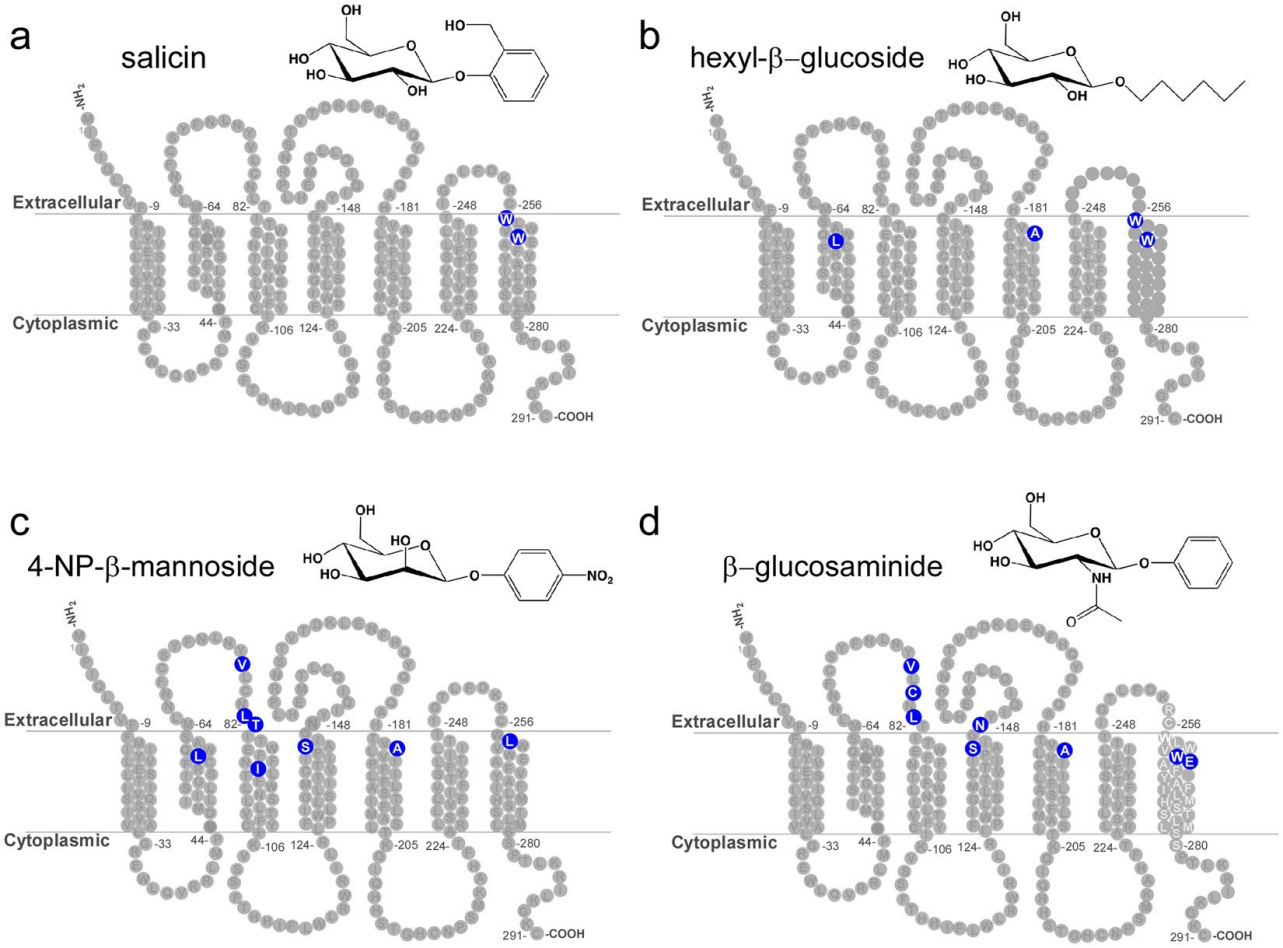
TAS2R16 residues that define ligand specificity. (**a–d**) Diagrams of TAS2R16 showing the locations of residues where mutations gave high activation with at least one ligand, low activation with another ligand, and >2.5-fold differential response among ligands. Each panel shows residues (in blue) where a mutation resulted in decreased activation by the specific ligand.

To understand how structurally distinct ligands can similarly activate TAS2R16, 4-NP-β-mannoside, hexyl-glucoside, and β-glucosaminide were each screened against the entire TAS2R16 mutation library, as for salicin, allowing us to distinguish how each of the four ligands interacts with the receptor. Pair-wise comparisons between ligands for their Ca^2+^ flux activities identified critical mutations responsible for differential ligand responses (i.e. resulting in low activity with one ligand, but normal activity with another) (Fig. 3a). The inability of a ligand to signal with a particular TAS2R16 variant implies that the associated mutated residue contributes to recognition of that specific ligand. In total, 13 critical mutations demonstrated ligand-specific effects (≥2.5-fold difference in activity for at least one pair-wise ligand comparison) (Fig. 3b; Supplementary Table S3). All 13 identified residues are positioned on the extracellular side of the transmembrane domains or in the ECL loops (Fig. 4), consistent with the proposed location of ligand-binding sites of TAS2R receptors and other GPCRs^19^.

Since all ligand-specific variants traffic to the surface at near wild-type levels, and signal at near wild-type levels for at least one ligand, global misfolding of the variants can be discounted as a cause of decreased activation.

Five of the variants that displayed ligand-specific activation contained mutations to alanine or glycine (L59A, V77A, L81A, T82A, W261G), so their effects on signaling could be ascribed solely to the loss of a side-chain interaction. Eight of the remaining nine variants were more divergent side-chain changes, and are expected to either alter specific contacts of the receptor-ligand interaction (I90T, S144L, N148V, A184V, L258V, E262D) or potentially introduce deleterious (steric) effects on ligand interactions (C79R, W257R).

Those positions that were mutated initially to residues other than alanine (C79, I90, S244, N148, A184, W257, L258, W261, and E262), were individually mutagenized to alanine (and A184 to serine), and we then determined the reactivity of these mutant receptors with the 4 ligands tested previously, and with an additional compound, 4-nitrophenyl-glucopyranoside (4-NP-β-glucoside) (Figure S3). Generally, alanine at these positions resulted in responses similar to the previous mutations tested, confirming our screening results and suggesting that the side-chain at each of these positions is interacting with the ligand. Conspicuous exceptions were variants C79A, I90, and E262A. C79A showed near WT levels of activation with all ligands tested, greater than the C79R variant. This was particularly clear for the response to β-glucosaminide (90% of wild-type activation with C79A, 10% with C79R), suggesting that C79R disrupts the interaction with β-glucosaminide, presumably close to the sugar C2 position that is the major difference from salicin. It is unknown whether C79 forms a disulfide bond with either C69 on ECL1 or C256 on ECL3. E262A eliminated activation by all compounds tested, while E262D had shown decreases only with 4-NP-β-mannoside, and particularly with β-glucosaminide (Fig. 3).

**Figure 5:**
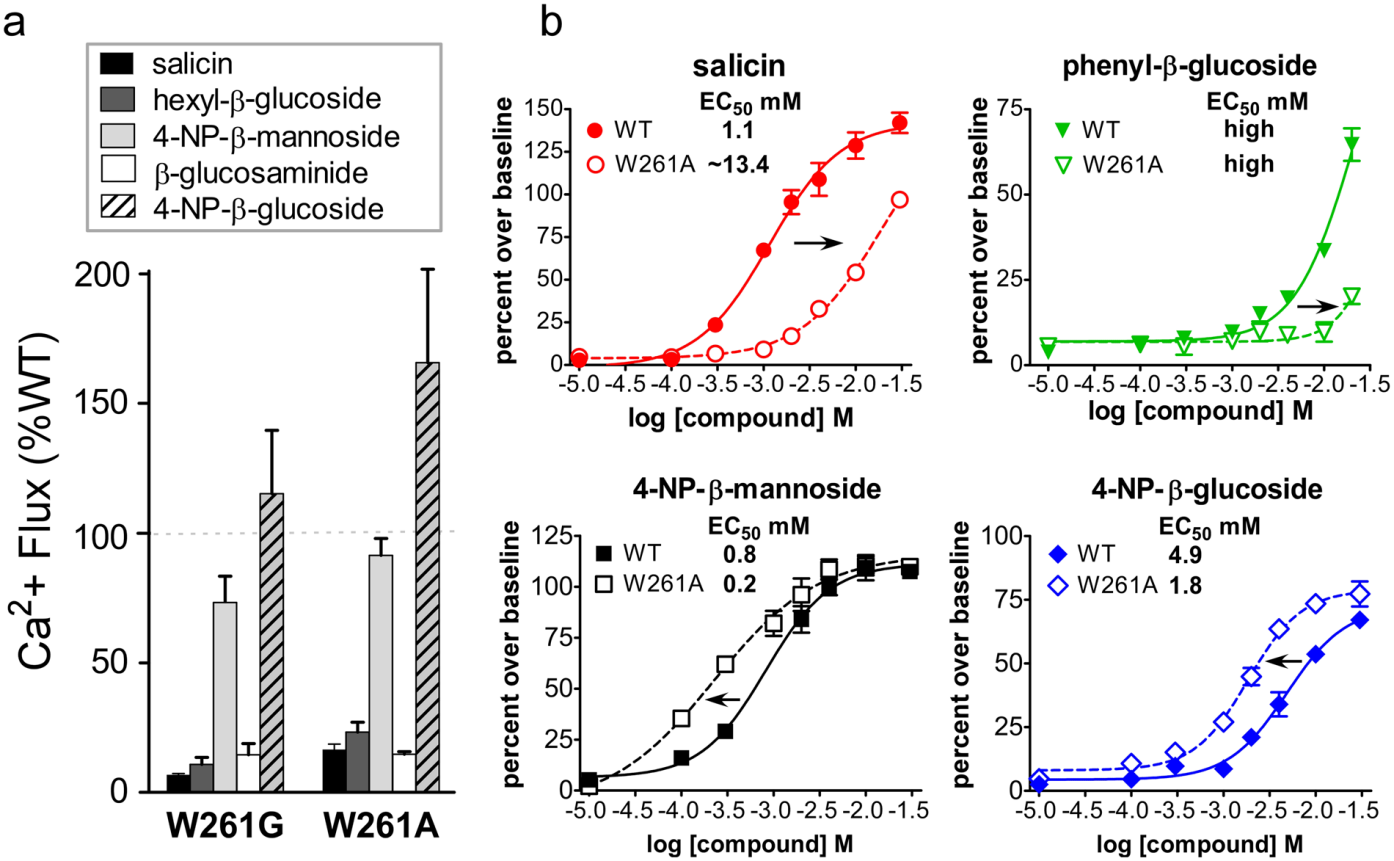
A mutation at TAS2R16 position 261 results in loss or gain of activity. (**a**) Activation of W261G and W261A TAS2R16 variants with a variety of ligands, activities shown as a % of their activation with wild-type TAS2R16. (**b**) Dose-response studies performed for wild-type (WT; solid symbols) and W261A TAS2R16 (open symbols) using the indicated ligands.

The W261A variant was strongly activated by 4-NP-β-mannoside and 4-NP-β-glucoside but showed almost no activity with the other three ligands tested (Fig. 5). While 4-NP-β-glucoside behaved similarly to salicin for some TAS2R16 mutants, for W261A it behaved more like 4-NP-β-mannoside.

### W261A results in ligand-dependent loss or gain of function

4-NP-β-mannoside has two substitutions that distinguish it from the other ligands tested here: an axial 2′-OH group rather than the equatorial 2′-OH of glucoside ligands, and a 4-nitrophenyl substitution on the phenolic R group. The ability of 4-NP-β-glucoside and 4-NP-β-mannoside alone to activate W261A TAS2R16 suggests that this activation is due to the 4-nitrophenyl moiety present in both ligands. It is notable that with W261G and W261A, 4-NP-β-glucoside results in activation levels higher than wild-type TAS2R16 (Fig. 5A).

We further examined the 261 position by using wild-type and W261A TAS2R16 for dose-response analyses with salicin, 4-NP-β-mannoside, phenyl-β-glucoside, and 4-NP-β-glucoside (Fig. 5B). With W261A, salicin and phenyl-β-glucoside showed increased EC_50_ values for activation (approximately 10-fold higher for salicin), relative to wild-type TAS2R16. In contrast, 4-NP-β-mannoside and 4-NP-β-glucoside had *decreased* EC_50_ values with W261A (i.e. stronger activation), showing that alanine at position 261 resulted in a gain-of-function for ligands with 4-nitrophenyl substitutions in the R-group, but loss-of-function with all other ligands tested. The 4-nitrophenyl substitution clearly exerts a major influence on ligand binding, and may enable different modes of binding, as suggested by the activation profile of the W261A mutant.

### Ligand substitutions influence TAS2R16 activation

**Figure 6:**
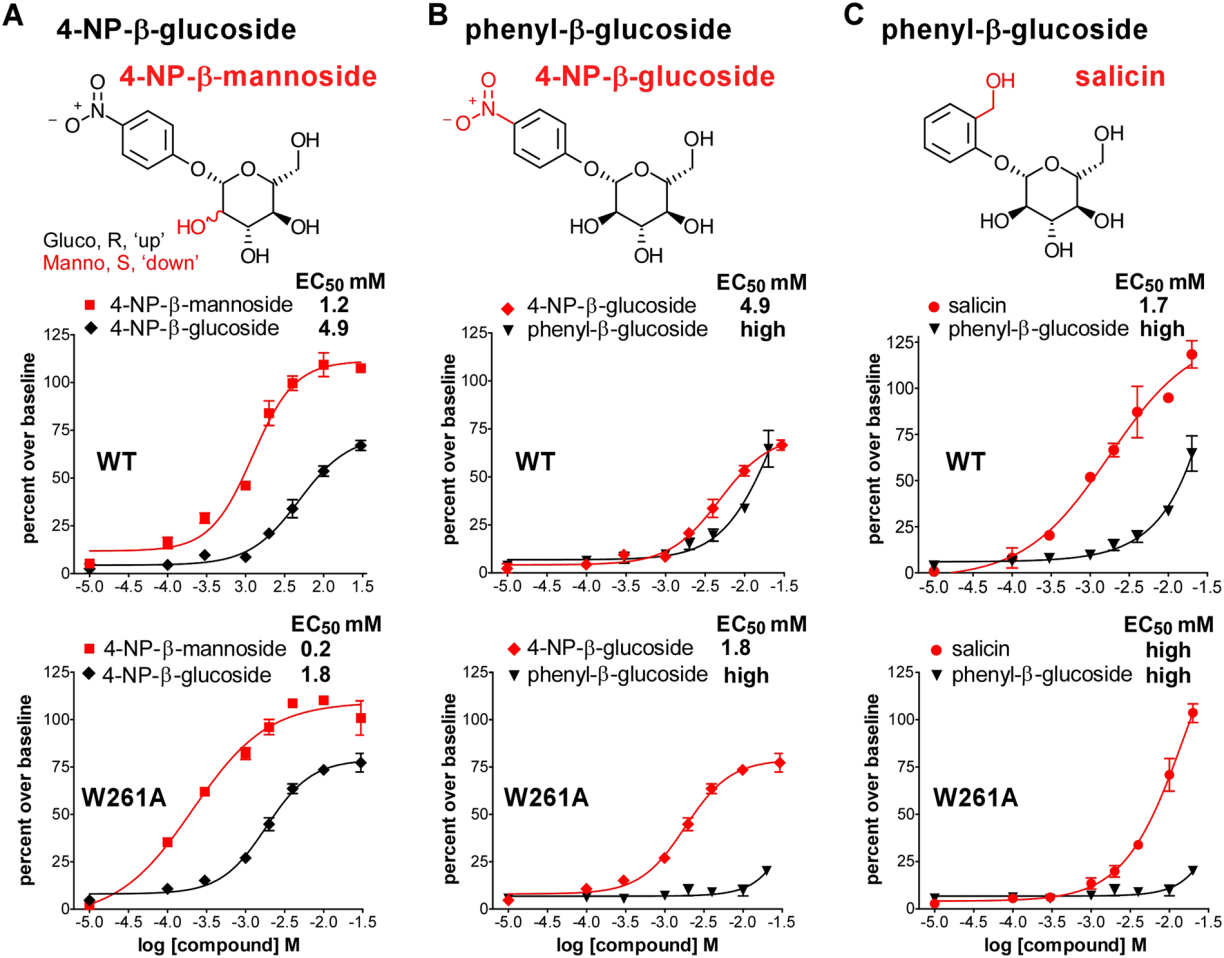
Specific ligand substitutions decrease the EC_50_ values for TAS2R16 activation. Comparative dose-response studies were performed with wild-type TAS2R16 and the W261A variant for ligand pairs (**a**) 4-NP-β-mannoside and 4-NP-β-glucoside, differing in the orientation of the 2′-OH group, (**b**) 4-NP-β-glucoside and phenyl-β-glucoside, and (**c**) salicin and phenyl-β-glucoside. Error bars represent the standard deviation, n = 4–8 replicate points.

A comparison of the dose-responses for TAS2R16 with different ligands demonstrated how specific substitutions within both the sugar and R group moieties affected the ability of ligands to activate wild-type TAS2R16 (Fig. 6).

### Sugar group 2′-OH orientation

4-NP-β-mannoside and 4-NP-β-glucoside differ solely by the orientation of the 2′-OH group on the sugar moiety. In dose-responses, 4-NP-β-mannoside, which has an axial 2′-OH group, had a 4-fold lower EC_50_ (Fig. 6A) than 4-NP-β-glucoside, which has an equatorial 2′-OH. 4-NP-β-mannoside also showed a higher maximal activation as compared to the corresponding glucoside. Both compounds show a decrease in EC_50_ value with W261A TAS2R16.

### R-group: 4-nitrophenyl substitution

4-NP-β-D-glucoside and phenyl-β-D-glucoside differ solely by the presence or absence of a 4-nitrophenyl substitution on the R group. A comparison of dose-response curves for these two ligands shows that the presence of a 4-nitrophenyl group results in both a decreased EC_50_ and a decreased extent of maximal activity **(**Fig. 6B). The W261A variant drastically decreased the activity of the parental phenyl-β-D-glucoside (higher EC_50_) while simultaneously increasing the activity (decreased EC_50_) of the 4-nitrophenyl-containing molecule.

### R-group: 2-hydroxymethyl substitution

Salicin and phenyl-β-D-glucoside differ solely by a 2-hydroxymethyl substitution on the salicin R group. Salicin displayed a considerably lower EC_50_ for TAS2R16 activation (Fig. 6C), suggesting that the 2-hydroxymethyl substitution confers a better fit for the binding pocket. Both compounds showed a decrease in activity with the W261A mutation.

### An inhibitor resistance mutation decreases ligand EC_50_

**Figure 7:**
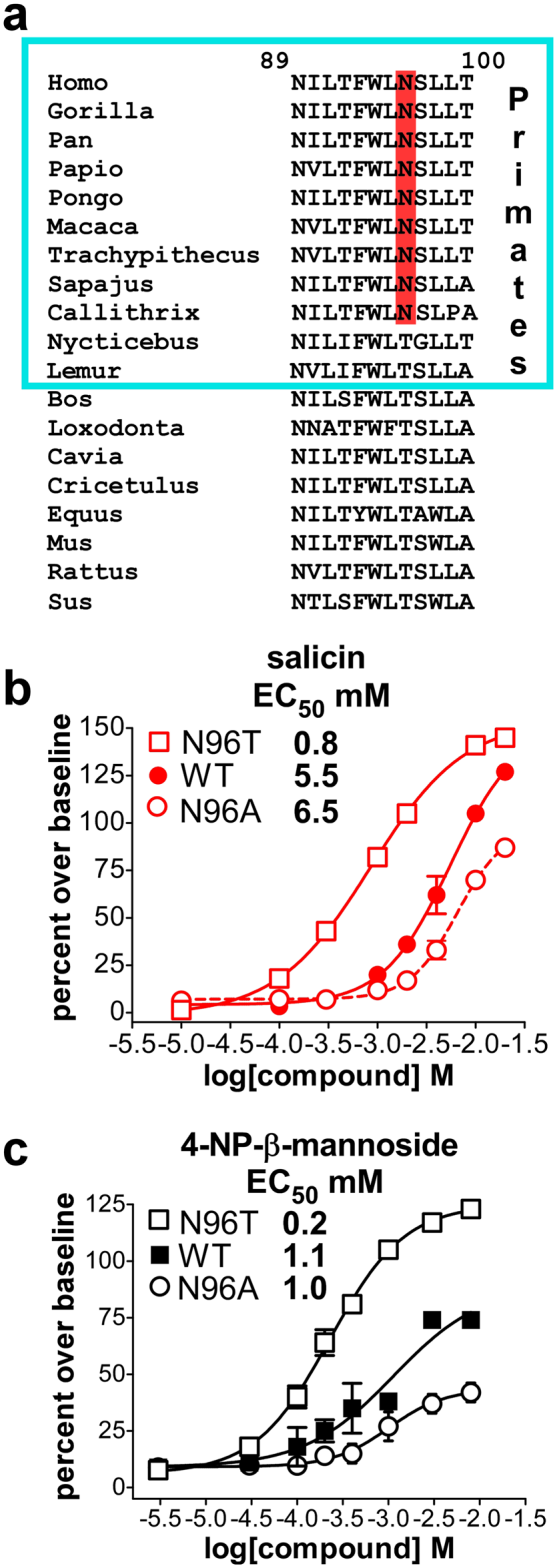
The N96T probenecid-resistance mutation decreases the EC_50_ for TAS2R16 activation. (**a**) Alignment of partial TAS2R16 sequences (corresponding to residues 89–100 in human TAS2R16) demonstrates that N96 (red highlight) replaced T96 during primate evolution. Shown are TAS2R16 sequences from *Gorilla gorilla* (western gorilla), *Pan troglodytes* (chimpanzee), *Papio anubis* (olive baboon), *Pongo pygmaeus* (Bornean orangutan), *Macaca mulatta* (rhesus macaque), *Trachypithecus cristatus* (silvery lutung), *Sapajus paella* (tufted capuchin monkey), *Callithrix jacchus* (marmoset), *Nycticebus coucang* (Sunda slow loris), *Lemur catta* (ring-tailed lemur), *Bos bovis* (cow), *Loxodonta africana* (African elephant), *Cavia porcellus* (guinea pig), *Cricetulus* *griseus* (Chinese hamster), *Equus caballus* (horse), *Mus musculus* (mouse), *Rattus norvegicus* (rat), and *Sus scrofa* (pig). Dose-response studies were performed for wild-type (WT), N96T, and N96A variants of TAS2R16 using ligands (**b**) salicin or (**c**) 4-NP-β-mannoside. Dose-response curves for salicin and 4-NP-β-mannoside with wild-type TAS2R16, and N96T and N96A variants demonstrated that N96T resulted in a greater than 5-fold decrease in EC50 for both compounds relative to wild-type TAS2R16. Error bars represent the standard deviation, n = 4–8 replicate points.

We showed previously that the compound probenecid acts as an allosteric inhibitor of TAS2R16, as well as TAS2R38 and TAS2R43^48^. We also previously identified mutation N96T in TAS2R16 as conferring resistance to probenecid^48^. A comparison of TASR16 sequences over a range of species showed that asparagine is found at position 96 only in higher primates. In other organisms, position 96 is occupied by threonine (Fig. 7A). This suggests that TAS2R16 function may be modulated by the nature of the specific amino acid present at position 96. To determine how the N96T mutation affected TAS2R16 function, we performed dose-response analyses on this variant. The N96T mutation decreased the EC_50_ for TAS2R16 activation by both salicin and 4-NP-β-mannoside by approximately 5-fold (Fig. 7B,C). The equivalence of the effect on both ligands suggests that this mutation acts independently of ligand type or is mediated by interactions conserved between the two molecules. Our data suggest that threonine at position 96 can result in greater sensitivity for TAS2R16 ligands.

### Identification of TAS2R16 residues required for signaling

**Figure 8:**
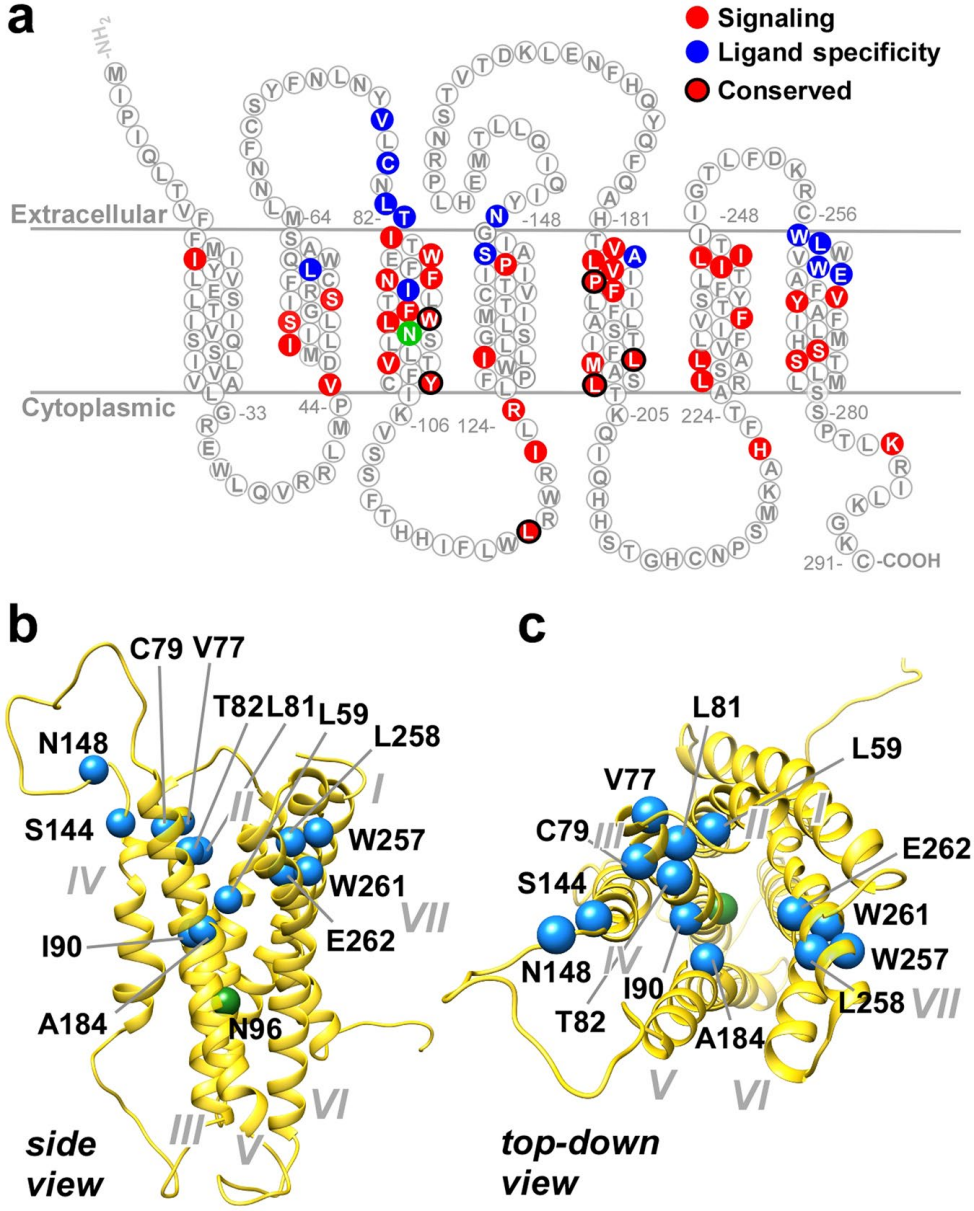
Summary of TAS2R16 residues critical for ligand binding and signaling. (**a**) TAS2R16 receptors with mutations at residues highlighted in blue signaled for at least one ligand, but failed to signal for another ligand. Residues highlighted in red failed to signal for all four ligands, but are expressed at wild-type levels on the cell surface. Signaling-critical residues that are highly conserved (>80% conservation among the 25 human TAS2R receptors) are circled in black. The N96 position which contributes to TAS2R16 probenecid sensitivity is shown in green. The residues implicated in ligand interactions (blue) are also shown on a TAS2R16 model structure (www.gpcrdb.org). The side view (**b**) suggests that the residues are located primarily in the membrane close to the extracellular side of the membrane, consistent with the locations identified in (**a**). TMs are identified by Roman numerals. The top-down view (**c**) suggests that the residues form a broad, two-sided ligand binding pocket.

The library screens identified residues responsible for ligand-specific activation, but also allowed us to determine common requirements for the four screened ligands. We identified 38 critical residues that were required for signaling by all four ligands (i.e. that fail to signal for all four ligands but that are expressed at wild-type levels at the cell surface) (Supplementary Table S1). Remarkably, these were identical to the 39 residues identified as critical for salicin signaling (Fig. 1C), except for residue W261, which was not required for activation by 4-NP-β-mannoside. All of the identified critical signaling residues are located in the TM helices or intracellular face of the receptor, consistent with a role in GPCR signal transduction. Although G protein coupling sites have not yet been defined for any TAS2Rs, a co-crystal structure of the β2-adrenergic receptor in complex with a G protein reveals that the receptor-G protein interface occurs primarily in the intracellular regions of TM3 and TM5^23^. Ten of the signaling residues are highly conserved (greater than 80% identity across human TAS2Rs) and seven of these are located within TM3 or TM5 (Fig. 8a, circled residues), consistent with potential roles in signal transduction and/or G protein coupling. Although local folding or structural defects cannot be definitively ruled out for all of these mutants, each clone was expressed at wild-type levels on the cell surface, a cellular quality control mechanism for most GPCRs^33^.

We cannot definitively determine whether these residues are involved in binding of all four ligands, signal transduction through the membrane, or with coupling to G proteins. Since studies of bitter taste receptors commonly employ a chimeric G protein forcing signaling through calcium, there is therefore a possibility that the structure-function relationships we have elucidated could differ slightly from those occurring with native G proteins such as gustducin.

There are currently no crystal structures for any TAS2Rs. Therefore, to visualize the critical residues that form the TAS2R16 binding pocket, we mapped the residues critical for ligand specificity on a TAS2R16 structural homology model (Fig. 8b) made available by the GPCR consortium (gpcrdb.org^34^). These results suggest a model in which β-glycoside ligands bind an unusually broad pocket exposed on the extracellular side of TAS2R16 (Fig. 8a,b, blue residues) and mediate signal transduction through the GPCR via conserved residues in the TMs and on the intracellular side of TAS2R16 (Fig. 8a, red residues).

## Discussion

### Determinants of TAS2R16 ligand interactions

The new TAS2R16 agonists identified here demonstrate that the TAS2R16 binding site can accommodate a wide variety of hydrophobic ligand R groups, including aromatics (both substituted and unsubstituted), aliphatics, and charged moieties, suggesting a large binding site capable of supporting diverse interactions. The activation by thioglucosides (phenyl-β-D-thioglucopyranoside and sinigrin) and disaccharides (gentiobiose and amygdalin)^4, 6^ is also consistent with a large binding site. In particular, a thioglycosidic linkage (substitution of oxygen by sulfur) has a significantly longer bond length than that of a standard β-glucoside (1.8 Å for C-S versus 1.4 Å for C-O), suggesting a binding site capable of supporting widely different R group orientations in the binding pocket. There do appear to be certain size restrictions for TAS2R16 ligands, with very small (methyl-) or very large (naphthyl-) R groups showing little to no receptor activation, and glucose itself (i.e. no R group) neither activating TAS2R16 nor being perceived as bitter.

For bitter taste receptors in general, and TAS2R16 in particular, substrates often exhibit high EC_50_ values, matching the natural levels of bitter toxins in food and implying weak (but specific) binding interactions. Despite the diversity of the tested TAS2R16 agonist structures, most fell within a narrow range of EC_50_ values (0.65 to 4.8 mM), suggesting that the strength of the overall interaction is determined primarily by the carbohydrate moiety, the structural element common to all ligands. However, the unsubstituted phenyl-β-glucoside showed substantially weaker activation than either salicin (ortho substitution with a hydroxymethyl group) or 4-NP-β-glucoside (Fig. 6), suggesting that a larger (substituted) R group is required for optimal ligand binding (affinity), receptor activation (efficacy), or both.

### TAS2R16 binding selectivity

Our screens of the TAS2R16 mutation library with four structurally-related agonists identified 13 residues that contribute to ligand-specific interactions, as well as 38 residues, throughout the transmembrane domains, that may be involved in mediating signal transduction. Since we have used a chimeric G protein forcing signaling through calcium, there is a possibility that the structure-function relationships we have elucidated could differ slightly from those occurring with native G proteins such as gustducin.

The most striking finding is the role of W261 in determining the specificity of glycoside ligands. Mutation of W261 to alanine or glycine led to a large loss of activity (>10-fold increase in EC_50_ with salicin, compared to WT) for salicin, hexyl-β-glucoside, β-glucosaminide, and phenyl-β-glucoside, but resulted in substantial *increases* in the activities of the two ligands with 4-nitrophenyl R groups, 4-NP-β-mannoside and 4-NP-β-glucoside (~4-fold decrease in EC_50_ compared to WT) (Figs 5 and 6). With loss of the tryptophan indole sidechain, a concomitant loss of function (as seen for salicin) could be ascribed to a loss of an interaction, but our results suggest that the removal of the indole group also enables additional (positive) interactions with 4-nitrophenyl-containing ligands. We propose that the R-group 4-nitrophenyl allows a distinct mode of ligand interaction with TAS2R16, but only upon removal of the bulky tryptophan at residue 261, in effect compensating for the loss of the indole interaction. A potential mechanism for this interaction is a 4-nitrophenyl binding site that is created by the loss of W261. Alternatively, loss of the tryptophan may allow the 4-nitrophenyl-glycoside ligand to reorient and make additional energetically favorable interactions with other parts of the receptor.

W261 is located in TM7, and several nearby TM7 residues identified in our screens (W257, L258, and E262) likely contribute interactions to the glycoside R group, with E262 of particular interest. E262A eliminated activation by all compounds tested (Figure S3), but E262D decreased activity solely for 4-NP-β-mannoside and phenyl-β-glucosaminide (Fig. 3). Given that both these ligands differ from the other tested glycosides at the carbohydrate C2 position, we speculate that the critical polar residue E262 contributes to interactions by hydrogen bonding to the ligand C2 moiety, which is especially relevant since C2-modified derivatives of salicin are often the majority bitter component found in plant materials^31^. Taken together, our results suggest that the TAS2R16 binding pocket can accommodate more complex natural products than previously thought, and that TM7 has a critical role in TAS2R16 ligand specificity.

The ECL1/TM3 regions also appear to contribute to ligand binding and, in particular, to modulating the interaction of TAS2R16 with 4-nitrophenyl-containing glycosides. Relevant residues include V77, L81, T82 and I90, whose hydrophobic nature is consistent with interactions with the hydrophobic R groups of ligands. The effect of mutations at these residues on activation by ligands containing the 4-nitrophenyl group can be interpreted as either a response to the decreased hydrophobicity of the R group, or to steric considerations due to reorientation of the ligand within the binding pocket.

Several other mutations showed differential effects on ligands (Figure S3). Activation by β-glucosaminide was particularly sensitive to mutations at residues C79 in TM3 and S144 and N148 in TM4, likely revealed due to the ligand's relatively low affinity for TAS2R16. Finally, we hypothesize that residues L59 and A184 also help define the size of the binding pocket for the glycoside R group. These residues lie deep in the pocket on the TAS2R16 homology model (Fig. 8). Mutation of L59 had the greatest effect on activation by hexyl-β-glucoside (with an extended R group) and 4-NP-β-mannoside and 4-NP-β-glucoside (which both have a nitrophenyl R group), suggesting that L59 lies at the edge of the pocket and that smaller amino acid substitutions can constrain the size of the R group permitted in the binding pocket. Similarly, mutation of A184 greatly decreased signaling by all ligands with the exception of the smallest ligand tested, salicin, suggesting that larger residues at this position limit the size of the binding pocket.

The mutation of asparagine 96 to threonine decreases the EC_50_ of both salicin and 4-NP-β-mannoside. However, residue 96, because of its location near the cytoplasmic end of TM3, is probably not directly involved in ligand interactions. Residue 96 lies further towards the cytoplasmic side of the receptor than residues shown to contribute to ligand specificity (Fig. 8). We speculate that the N96T-associated decreases in EC_50_ (higher sensitivity) of salicin and 4-NP-β-mannoside are due to changes in the relative stability of active and inactive forms of the GPCR. This would correspond with residue 96 acting as a gain switch for the GPCR. Residue 96 is asparagine in most primates, except for lemurs and lorises which, like other mammals, have a threonine in this position, predicted by our data to result in a more sensitive receptor than the N96 variant. Thus, we would predict that primates, except for lemurs and lorises, have a lower sensitivity to plant glycosides. Changes in taste receptor sensitivity are related to changes in diet, possibly reflecting the more generalist (and frugivorous) primate diet. This is consistent with TAS2R16 being under active selection^28, 35^. We also note that many carnivores do not have a functional TAS2R16^36, 37^.

### A model of TAS2R16 binding

Our data suggest a model of TAS2R16 in which a large ligand-binding pocket with critical energetic contributions from residues in TM3 and TM7 allows the sampling of distinct glucose and mannose stereochemistries to provide broad substrate reactivity. The large binding pocket enables diverse glycoside structures to fit while limiting the number of high-affinity interactions that can occur simultaneously. It is notable that W261A eliminated activation by salicin but *increased* the activation by 4-NP-β-mannoside and 4-NP-β-glucoside, suggesting that the mutation enabled an altered interaction between these ligands and the binding pocket.

It is clear that TM3 and TM7 of TAS2R16 play a key role in receptor specificity and activation. The role of TM3 and TM7 in GPCR function is well understood^24^. In particular, a number of GPCR structures show TM3 and TM7 being bridged by agonist, for example, the β2-adrenergic receptor (β2AR), purinergic receptor P2Y12, and µ-opioid receptor^38–40^. Our results enable us to propose a model for the TAS2R16 ligand-binding pocket where specific interactions of the ligand R group (or potentially the carbohydrate moiety) bridge residues in TM3 and TM7 leading to a conformational change and subsequent activation of the receptor. This model is consistent with the ligand- and mutation-dependent changes that we observed in both ligand EC_50_ and maximal activation.

While a handful of the individual residues that we identified as critical have previously been postulated to be important for TAS2R signaling (discussed below), our work extends these earlier results to explain how the TAS2R binding pocket accommodates structural diversity while maintaining high specificity. A previous study of TAS2R16 proposed a model for the binding pocket after determining the effect of eight residue mutations on activation by salicin and two closely-related agonists^6^. We did not identify any of these residues as being involved in specific ligand binding, but four (N89, F93, W94, and I243) are among the 38 residues that we identified as critical for signaling by all four ligands, and mutations at two other residues (E86 and F240) reduced signaling by all ligands (although neither reduced salicin signaling below the cutoff value we used to determine critical residues). Mutation of the two other residues, Q177 and H181, had little effect on signaling by any of the ligands tested here, suggesting that they are not critical for TAS2R16 activity.

### Application of the model to other TAS2Rs

Some residues identified here as important for ligand interaction and receptor function are also important for the function of diverse TAS2Rs. Residues equivalent to N89, E262, V265, and S275, identified here as critical for activation of TAS2R16, have also been identified as important for activation in other TAS2Rs^16, 41–45^. Such similarities suggest that the model proposed here for TAS2R16 function may be broadly applicable across the TAS2R family, all of which face similar challenges in recognizing diverse structural features while maintaining specificity. The conservation of many of the identified signaling residues, particularly those in TM3 and TM5, is also consistent with these residues playing a common role in the signaling mechanism of all bitter taste receptors.

It is interesting to note that eight of the critical TAS2R16 residues identified here (I13, V77, R124, N148, L185, M200, W261, S273) are conserved between humans and guinea pigs (which have similar bitter taste sensitivity to salicin) but not in mice, which are indifferent to glucopyranosides but become responsive when expressing human TAS2R16 as a transgene^46, 47^. This suggests that the ligand specificity of murine TAS2R16 may be significantly different than that of the human receptor. Thus, our results may provide a structural explanation for how different animals perceive the bitter taste of plant glycosides and define their diet.

## Conclusion

Using a comprehensive library of single amino acid mutations covering all 291 residues of TAS2R16, we have identified 13 TAS2R16 residues that contribute to ligand-specific interactions and 38 residues that may mediate signal transduction, providing a comprehensive assessment of how this GPCR binds and signals. In particular, we show a key role for TM3 and TM7 in determining the function of TAS2R16. Gain-of-function mutations at several sites indicate the plastic nature of the ligand-binding site and the potential for natural polymorphisms to substantially alter ligand specificity and receptor activity. We expect that our model can be further refined in future studies, including using additional TAS2Rs and structures. This structural information may also be useful for the synthetic design of new, high affinity TAS2R ligands that could be used as bitter blockers to improve food choice and medication compliance.

## Methods

### Preparation of TAS2R16 Mutation Library

A TAS2R16 eukaryotic expression vector was constructed as described previously^48^, encoding full-length TAS2R16 with an N-terminal FLAG epitope tag, an SST3 signal sequence, and a C-terminal V5 epitope tag. Using this TAS2R16 expression construct as a template, a library of random mutations (Diversify PCR Random Mutagenesis Kit, Clontech) was created using a PCR-based “shotgun mutagenesis” method so that every amino acid position was mutated^21, 49^. Each mutant clone was fully sequenced, and 21 residue changes not obtained by random mutation were changed to alanine by site-directed mutagenesis (QuikChange, Agilent Technologies). Clones exhibiting one to three substitutions in the TAS2R16 coding region were selected to create a comprehensive mutation library comprised of 573 mutant TAS2R16 plasmids with substitutions in all 291 residues.

### Immunofluorescence assays

Each construct in the mutation library was tested for full-length TAS2R16 translation and surface expression by immunofluorescent antibody binding assays. The mutation library and controls were expressed in HEK-293T cells in 384-well plates as described^21^. Twenty-four hours post-transfection, cells were washed with PBS-/- (HyClone), followed by addition of cell stripper (Cellgro). Suspended cells were fixed with paraformaldehyde at a final concentration of 4%. To detect surface expression of proteins, cells were incubated for one hour with anti-FLAG MAb M2 (1:500; Stratagene). To determine total (full-length) expression, cells were permeabilized using PBS++ (HyClone) with 0.1% saponin and incubated with an anti-V5 antibody (Invitrogen R960-25) for one hour. Primary antibody incubations were followed by a one hour incubation with goat anti-mouse Cy3-conjugated secondary antibody (1:500; Jackson Laboratories). Secondary antibody fluorescence was measured from a minimum of 500 cells by flow cytometry on an Intellicyt HTFC screening system. FLAG and V5 reactivities for library clones were normalized to the values obtained from wild-type TAS2R16.

### Calcium flux assays

For initial screens, the following compounds (all from Sigma, St. Louis, MO unless indicated) were used in Ca^2+^ flux assays with HEK-293T cells expressing wild-type TAS2R16, as previously described^48, 50^: salicin; phenyl-β-D-thioglucopyranoside; phenyl-β-D-glucopyranoside (Carbosynth, Berkshire, United Kingdom); hexyl-β-D-glucopyranoside (Carbosynth); phenyl-N-acetyl-β-D-glucosaminide; sinigrin; 2-naphthyl-β-D-gluco-pyranoside; esculin; methyl-β-D-glucopyranoside; phenyl-α-D-glucopyranoside; 1-O-phenyl-β-D-xyloside; phenyl-β-D-galactopyranoside; 4-nitrophenyl-β-D-mannopyranoside; phenyl-α-D-glucopyranoside; or hexyl-β-D-glucopyranoside. The TAS2R16 mutation library was tested for function using a Ca^2+^ flux assay as described previously^48^. Later screens of individual TAS2R16 mutant clones used 4-nitrophenyl-β-D-glucopyranoside. Briefly, HEK-293T cells, in poly-lysine coated, black 384-well plates with clear bottoms (Costar), were transfected with the TAS2R16 mutation library, and a plasmid expressing a Gα16 chimera containing the last 44 amino acids of rat gustducin (Gα16gust44). Cells were incubated for 22 hours at 37 °C then washed twice with a calcium indicator dye in HBSS containing 20 mM HEPES (Calcium 4 Assay kit, Molecular Devices), incubated with HBSS for 1.5 hr, then moved to a Flexstation II-384 (Molecular Devices) set at 32 °C. Probenecid, a commonly used additive used to improve dye-loading of cells, was not included during the incubation due to our previous demonstration of probenecid as a TAS2R16 inhibitor^48^. After a 10-minute temperature equilibration, one of the following ligands was injected (at t = 20 seconds) to the final concentration indicated and fluorescence was measured for 60 seconds (reading every 3 seconds): 3 mM salicin, 5 mM hexyl-beta-D-glucoside, 1.6 mM 4-nitrophenyl-β-D-mannopyranoside, or 10 mM phenyl-N-acetyl-β-D-glucosaminide. Concentrations for mutation library screening were chosen to be approximately 2 to 3 times higher than the EC_50_ values for the ligands to maximize the sensitivity of detecting decreases in signaling due to the mutation in each clone.

### Data Analysis

For Ca^2+^ flux assays, the value of fluorescence at 30 seconds after ligand addition (the time of peak signal for wild-type TAS2R16) was used as the maximum value. The ligand phenyl-N-acetyl-β-D-glucosaminide exhibited slightly slower activation kinetics, so the fluorescence value at 39 seconds was used. Raw data obtained from surface expression and flux experiments were normalized to the values for wild-type TAS2R16 on each plate. Data sets were analyzed and represented as percentage over baseline signal. Mean and standard deviation values were calculated for each clone from replicate assay data. To identify critical residues involved in ligand-induced signaling, clones were selected that showed Ca^2+^ flux below the value of (average negative control +3*SD), but with surface expression above a threshold value for FLAG of (100-3*SD of the mean wild-type control wells on each plate).

To identify clones that demonstrated Ca^2+^ fluxes with differential responses to ligands, clones were selected that gave Ca^2+^ flux greater than (100-3*SD from positive control wells) for one ligand, but less than (100-3*SD from positive control wells) for the compared ligand. Clones were selected for further screening if they exhibited ligand activation values 2.5-fold higher or lower than those obtained with the compared ligands.

Ligand dose-response data was analyzed with Graphpad Prism 5 software. Data was baseline subtracted and then fit using a sigmoidal dose-response (variable slope) equation.

## Electronic supplementary material

Supplementary Information
