# Supplementary material for: The Bitter Taste Receptor TAS2R16 Achieves High Specificity and Accommodates Diverse Glycoside Ligands by using a Two-faced Binding Pocket
Source: Sci Rep. 2017 Aug 10;7:7753. doi: 10.1038/s41598-017-07256-y (PMC5552880; doi:10.1038/s41598-017-07256-y)
Supplement: Supplementary file 1 — Supplementary Information [file 41598_2017_7256_MOESM1_ESM.pdf]

SUPPLEMENTARY INFORMATION

for

The Bitter Taste Receptor TAS2R16 Achieves High Specificity and Accommodates Diverse Glycoside Ligands by using a Two-faced Binding Pocket

Anu Thomas, Chidananda Sulli, Edgar Davidson, Eli Berdougo, Morganne Phillips, Bridget A. Puffer, Cheryl Paes, Benjamin J. Doranz, and Joseph B. Rucker*

Integral Molecular, Inc. 3711 Market St, Suite 900, Philadelphia, PA 19104 USA

*Corresponding author: jrucker@integralmolecular.com

Location	Mutation	Ca^{2+} Flux Sal	Mann	Hexyl	Glucos	Total Expression	Surface Expression	% TAS2R identity
TM1	I13N	5 (5)	2 (8)	2 (1)	3 (6)	118 (33)	80 (10)	8
TM2	V45E	2 (4)	3 (6)	3 (7)	2 (8)	99 (48)	126 (11)	20
TM2	I48S	10 (6)	8 (6)	6 (0)	8 (5)	110 (56)	86 (34)	88
TM2	S51R	9 (4)	10 (7)	6 (3)	9 (11)	84 (37)	63 (18)	24
TM2	S55F	11 (2)	6 (9)	5 (3)	1 (4)	75 (12)	94 (20)	84
TM3	I83N	10 (19)	0 (7)	4 (5)	3 (4)	110 (40)	127 (12)	12
TM3	W85R	-2 (7)	-2 (5)	-2 (2)	0 (11)	94 (27)	99 (15)	84
TM3	F88V(S108T)	4 (3)	-1 (8)	2 (0)	2 (5)	101 (28)	112 (24)	4
TM3	N89I	2 (2)	3 (4)	1 (4)	3 (4)	104 (26)	115 (10)	84
TM3	F93I	1 (3)	-1 (9)	1 (2)	7 (2)	94 (60)	123 (41)	8
TM3	W94G	3 (3)	1 (3)	-1 (5)	2 (3)	97 (18)	118 (37)	100
TM3	L95S	2 (3)	-2 (6)	2 (6)	2 (8)	107 (23)	107 (8)	56
TM3	V101E	7 (11)	4 (8)	5 (2)	5 (6)	72 (1)	84 (14)	32
TM3	Y103H	8 (11)	5 (5)	3 (3)	7 (6)	90 (37)	80 (26)	92
ICL2	L118Q	6 (5)	3 (14)	11 (3)	9 (3)	68 (17)	100 (15)	88
ICL2	I122S	7 (15)	5 (7)	5 (2)	6 (4)	93 (38)	107 (34)	36
ICL2	R124W	7 (8)	4 (13)	5 (4)	13 (10)	85 (11)	116 (22)	16
TM4	I129K	-2 (5)	0 (1)	-2 (5)	3 (5)	96 (13)	89 (17)	40
TM4	P143H	3 (5)	2 (1)	-1 (1)	1 (6)	107 (49)	106 (7)	12
TM5	V183D	2 (4)	3 (2)	-1 (4)	1 (5)	98 (16)	86 (19)	16
TM5	L185S	6 (2)	7 (10)	4 (12)	5 (15)	111 (36)	128 (8)	4
TM5	V186D	2 (8)	3 (11)	-1 (6)	14 (22)	122 (18)	108 (0)	12
TM5	P188S(S228T)	9 (5)	2 (11)	3 (24)	5 (17)	101 (20)	163 (5)	92
TM5	F189S	5 (4)	3 (1)	-2 (3)	4 (11)	136 (17)	98 (2)	72
TM5	L199R	2 (7)	-1 (12)	-1 (8)	2 (9)	132 (45)	104 (14)	100
TM5	M200R	2 (2)	0 (4)	4 (6)	10 (15)	108 (3)	96 (4)	4
TM5	L203R	0 (4)	3 (3)	1 (6)	-3 (11)	102 (9)	95 (7)	96
TM6	L226Q	7 (3)	4 (4)	6 (7)	6 (16)	129 (8)	134 (8)	64
TM6	L229H	4 (2)	5 (4)	5 (13)	8 (7)	120 (4)	116 (23)	28
TM6	F236Y	2 (13)	2 (11)	0 (8)	6 (5)	104 (32)	122 (32)	36
TM6	I243T	10 (4)	2 (9)	4 (6)	6 (7)	91 (31)	100 (2)	20
TM6	L244R	2 (1)	3 (7)	-1 (5)	19 (33)	128 (10)	69 (4)	24
TM6	I245N	2 (8)	1 (3)	0 (2)	5 (5)	107 (14)	71 (14)	56
TM7	V265A	5 (4)	7 (13)	2 (13)	6 (8)	94 (2)	121(15)	24
TM7	Y266N	2 (6)	-4 (6)	0 (6)	9 (16)	101 (26)	94 (3)	12
TM7	S273F	8 (7)	3 (6)	-2 (8)	8 (13)	N/A	124 (2)	72
TM7	S275T	5 (5)	-1 (4)	5 (8)	24 (30)	107 (14)	122 (17)	4
C	K284E	7 (4)	10 (6)	5 (12)	4 (8)	102 (3)	129 (10)	56

Supplementary Table S1. TAS2R16 mutations critical for signaling induced by all four ligands. Mutations that abrogated agonist-dependent signaling were identified by screening a TAS2R16 mutation library for Ca^{2+} flux activity. Shown are the mutations that eliminated Ca^{2+} flux activity (< mean of negative controls + 3*SD, with flux measured as a % of wild-type) for salicin (Sal), 4-nitrophenyl-β-D-mannopyranoside (Mann), hexyl-β-D-glucopyranoside (Hexyl), and phenyl-N-acetyl-β-D-glucosaminide (Glucos) and their locations in the predicted structural feature (TM, transmembrane domain; ICL, intracellular loop). Total expression of TAS2R16 was measured by V5 expression and surface expression was measured by FLAG expression, each shown as a percentage of wild-type. Values indicate the mean (and range, max-min) of replicate measurements (n=3). Percent identity (%TAS2R) indicates the identity of the original TAS2R residue among all 25 members of the human TAS2R. F88V and P188 were screened as double-mutants (F88V, S108T) and (P188S, S228T), and the non-contributing mutations and their signaling values were S108T (99, 103, 122, and 132% of wild-type respectively, for the four ligands) and S228T (90, 120, 122, and 105 % of wild-type respectively, for the four ligands).

Location	Mutation	Ca^{2+} Flux	Total Expression	Surface Expression	% TAS2R identity
TM1	Y14H	9 (9)	66 (10)	33 (11)	4
TM1	I21F	9 (5)	91 (48)	36 (3)	4
TM1	Q24R	9 (9)	85 16)	30 (6)	4
TM2	D46A	8 (9)	89 (5)	45 (26)	84
TM2	R56P	2 (1)	95 (32)	46 (18)	96
TM2	C58R	5 (2)	98 (25)	48 (7)	24
TM2	L59P	2 (3)	75 (52)	36 (10)	92
TM2	Q60R	4 (7)	110 (32)	41 (12)	28
TM2	W61R	15 (7)	75 (12)	32 (3)	52
ECL1	M64R	14 (1)	80 (26)	34 (3)	56
TM3	S97R	0 (5)	91 (2)	40 (6)	12
TM3	C104R	9 (1)	66 (20)	16 (3)	36
TM4	L134R	1 (8)	83 (30)	30 (5)	42
**ECL2**	**N172K**	**83 (13)**	**95 (38)**	**48 (14)**	**8**
TM5	L191R	1 (3)	93 (14)	29 (7)	42
TM7	M271R	7 (6)	116 (11)	41 (5)	4
TM7	L276R	17 (7)	86 (10)	30 (8)	96

Supplementary Table S2. Mutations that diminished trafficking of TAS2R16 to the cell surface. Shown are the mutations that decreased trafficking of TAS2R16 to the cell surface, along with their ability to induce Ca^{2+} flux in response to salicin, total expression, and surface expression, all indicated as the mean (and range, max-min) of replicate (n=3) measurements compared to wild-type. For each mutation, its location in the predicted structure (ECL, extracellular loop; TM transmembrane domain) and conservation of original residue over the 25 members of the human TAS2R family (%TAS2R identity) is shown. Data for N172K, a naturally occurring polymorphism, is highlighted in bold.

Mutation	Location	Surface Expression	Ca^{2+} Flux Salicin	β-Manno-pyranoside	Hexyl-gluco-pyranoside	β-Glucos-aminide	% TAS2R identity
L59A	TM2	129 (41)	64 (15)	15 (16)	8 (5)	46 (9)	92
V77A	ECL1	103 (23)	84 (24)	30 (31)	58 (13)	30 (19)	12
C79R	ECL1	110 (28)	34 (17)	78 (4)	63 (18)	10 (12)	8
L81A	ECL1	155 (18)	92 (21)	12 (14)	59 (27)	15 (3)	12
T82A	ECL1	81 (12)	94 (22)	22 (8)	57 (19)	106 (29)	12
I90T	TM3	104 (32)	76 (41)	20 (19)	53 (26)	70 (19)	4
S144L	TM4	113 (19)	67 (41)	19 (3)	54 (9)	16 (17)	4
N148S	ECL2	103 (23)	79 (14)	73 (19)	81 (7)	30 (32)	12
A184V	TM5	114 (7)	88 (2)	12 (13)	28 (2)	31 (32)	36
W257R	TM7	120 (12)	26 (6)	68 (17)	18 (14)	30 (20)	12
L258V	TM7	97 (29)	37 (8)	8 (17)	26 (5)	48 (12)	8
W261G	TM7	86 (3)	11 (13)	88 (23)	12 (5)	7 (3)	12
E262D	TM7	82 (14)	92 (15)	40 (10)	90 (12)	22 (30)	8

Supplementary Table S3. TAS2R16 residues critical for activation by specific agonists. Calcium flux activities (shown as a % of wild-type activity) are shown for TAS2R16 clones with mutations at residues that were critical for Ca^{2+} flux for at least one, but not all, of the four ligands. Also shown are the locations in the predicted structure (ECL, extracellular loop; TM transmembrane domain; ICL, intracellular loop), and TAS2R16 surface expression (as a % of wild-type). Values indicate the mean (and range) of replicate measurements. %TAS2R identity indicates identity of the original residue compared to all 25 members of the human TAS2R family.

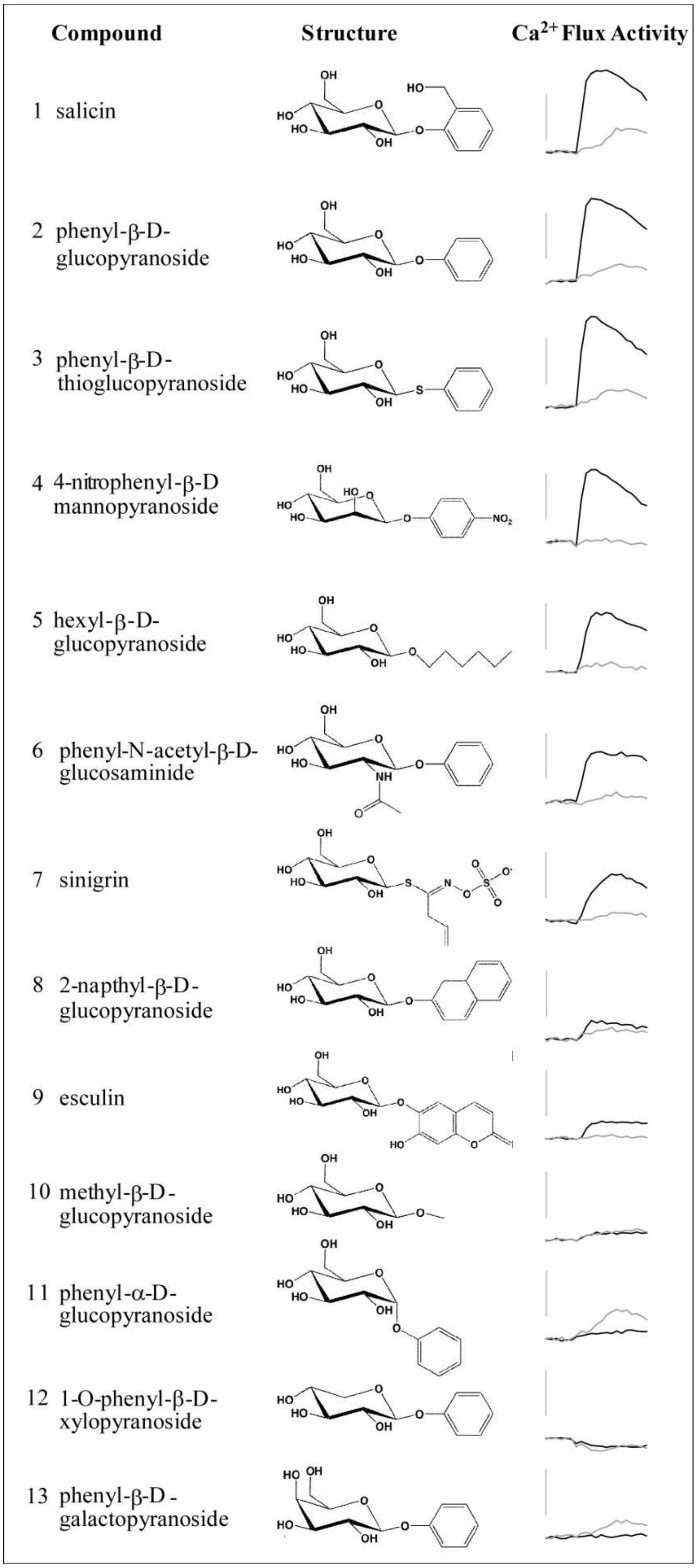

Supplementary Figure S1. Glycoside compounds tested for their ability to induce Ca^{2+} flux in cells expressing TAS2R16. Shown are representative Ca^{2+} flux traces (at identical scales, shown as relative fluorescence units) obtained from HEK-293T cells transiently transfected with wild type TAS2R16 and Gα16gust44, after addition of the indicated compound at 10 mM (black traces). Gray traces represent addition of compound to cells transfected with vector alone.

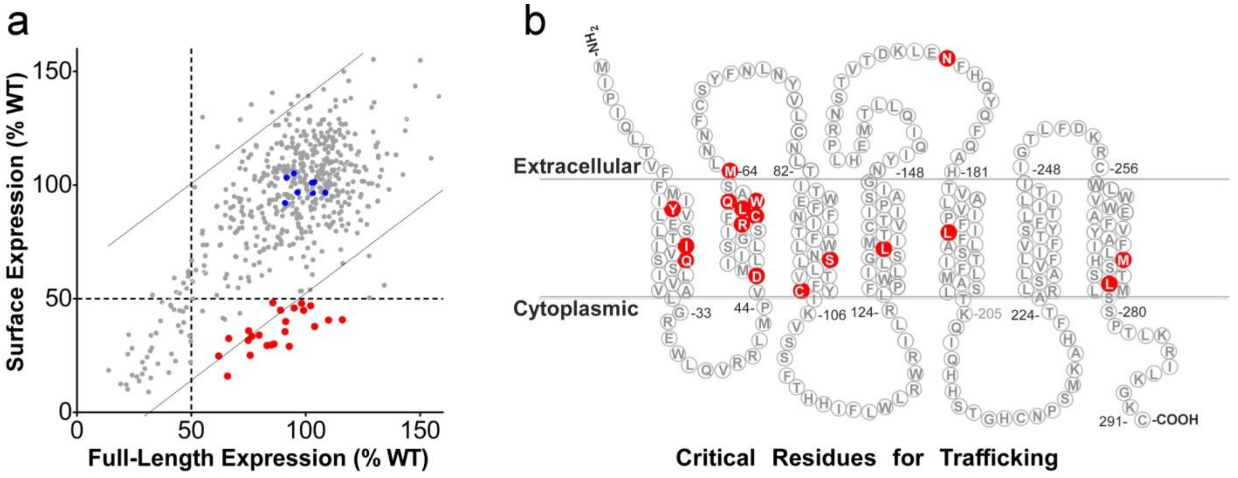

Supplementary Figure S2. Location of TAS2R16 trafficking mutants. (**a**) TAS2R16 was well expressed on the cell surface in the majority of the library clones, as detected by FLAG immunostaining. Clones deemed defective for trafficking (marked in red) were identified as those with >50% wild-type full-length expression (detected by V5 immunostaining), but surface expression <50% of wild-type, and being outside the 95% confidence intervals (diagonal lines). Average values obtained from wild-type controls are shown in blue. (**b**) Critical residues for surface trafficking (colored in red) are mapped onto a schematic diagram of TAS2R16.

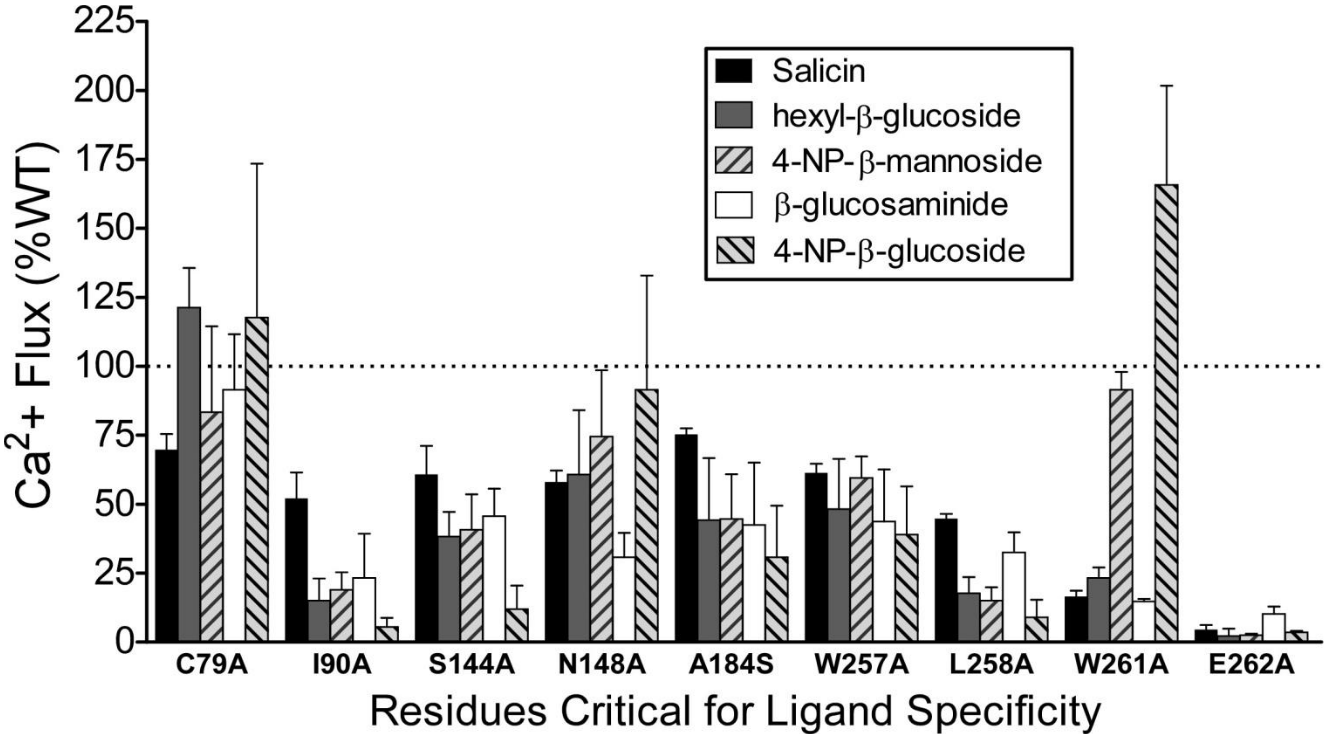

Supplementary Figure S3. Effect of side chain on ligand activation. Critical residues identified as ligand specific were changed to alanine and tested for their responses to the indicated ligands.
